# Structural Disconnection of the Tool Use Network after Left Hemisphere Stroke Predicts Limb Apraxia Severity

**DOI:** 10.1093/texcom/tgaa035

**Published:** 2020-07-28

**Authors:** Frank E Garcea, Clint Greene, Scott T Grafton, Laurel J Buxbaum

**Affiliations:** Moss Rehabilitation Research Institute, Elkins Park, PA 19027, USA; University of Pennsylvania, Philadelphia, PA 19104, USA; Department of Psychological and Brain Sciences, University of California at Santa Barbara, Santa Barbara, CA 93016, USA; Department of Psychological and Brain Sciences, University of California at Santa Barbara, Santa Barbara, CA 93016, USA; Moss Rehabilitation Research Institute, Elkins Park, PA 19027, USA; Department of Rehabilitation Medicine, Thomas Jefferson University, Philadelphia, PA 19107, USA

**Keywords:** connectome-based lesion-symptom mapping, dorsal stream, limb apraxia, tool use, voxel-based lesion-symptom mapping

## Abstract

Producing a tool use gesture is a complex process drawing upon the integration of stored knowledge of tools and their associated actions with sensory–motor mechanisms supporting the planning and control of hand and arm actions. Understanding how sensory–motor systems in parietal cortex interface with semantic representations of actions and objects in the temporal lobe remains a critical issue and is hypothesized to be a key determinant of the severity of limb apraxia, a deficit in producing skilled action after left hemisphere stroke. We used voxel-based and connectome-based lesion-symptom mapping with data from 57 left hemisphere stroke participants to assess the lesion sites and structural disconnection patterns associated with poor tool use gesturing. We found that structural disconnection among the left inferior parietal lobule, lateral and ventral temporal cortices, and middle and superior frontal gyri predicted the severity of tool use gesturing performance. Control analyses demonstrated that reductions in right-hand grip strength were associated with motor system disconnection, largely bypassing regions supporting tool use gesturing. Our findings provide evidence that limb apraxia may arise, in part, from a disconnection between conceptual representations in the temporal lobe and mechanisms enabling skilled action production in the inferior parietal lobule.

## Introduction

The ability to produce skilled object-directed action in order to satisfy behavioral goals is a cornerstone of high-level cognitive and motor function, referred to as praxis. A central focus in the study of human praxis function is to elucidate the cognitive and neural mechanisms that interface visual processing, semantic memory, and skilled action production in order to recognize and manipulate objects in a functionally appropriate manner. A point of entry into the study of object-directed action derives from neuropsychological assessments of patients with upper limb apraxia (hereafter referred to as apraxia). Apraxia is a deficit in producing and imitating gestures that is not reducible to low-level sensory or motor dysfunction (for review, see Johnson-Frey 2004). Patients with apraxia are impaired in producing skilled actions, often misshaping the fingers and hands when instructed to pantomime the use of objects or imitate novel gestures. Several “box and arrow” information processing models (e.g., see Roy and Square 1985; Rothi et al. 1991; Rumiati et al. 2010) posit a number of critical cognitive components that lead to different types of apraxia when lesioned or disconnected. It is only recently, however, that investigators have begun to consider how distributed cognitive models of praxis may be mapped to distributed neuroanatomic substrates (e.g., Watson et al. 2019). Understanding the consequence of specific lesion loci and disconnectivity for patterns of apraxia has implications for understanding the representation of action and object knowledge in the human brain, and provides a basis with which to identify the underlying structural connectivity interfacing object knowledge with action production processes to support skilled object-directed action.

Most cognitive models of praxis posit a distinction between a direct and indirect route for action processing (Rothi et al. 1991; Rumiati and Humphreys 1998; Cubelli et al. 2000; Buxbaum 2001). The direct route transforms current visual input into motor output, driven “bottom-up” by the stimulus. The indirect route supports gesture production by interfacing current visual input with stored action representations. The indirect route provides a processing advantage when generating a meaningful gesture (e.g., hammering a nail), because the implementation of that gesture is facilitated by semantic memory, including the retrieval of the visual appearance of a gesture (e.g., that a swung hammer moves in a particular trajectory), function knowledge retrieval (e.g., that the function of the act of hammering is to pound in nails), and the retrieval of postural knowledge (e.g., that the configuration of the hand and fingers is positioned in a nonarbitrary manner in order to functionally grasp and manipulate a hammer; e.g., see Bracci and Peelen 2013). In this regard, performance when gesturing tool use is a sensitive assay of the contributions of the indirect route, as the visual input must interface with the semantic system for accurate demonstration of tool use. By contrast, imitation of meaningless gestures provides a means with which to evaluate the integrity of the direct route, because the transformation of visual input into sensory–motor plans occurs without access to semantic information (for review, see Binkofski and Buxbaum 2013).

The left inferior parietal lobule is a core region supporting praxis function, as lesions to this region (supramarginal gyrus, angular gyrus) are associated with spatial and temporal errors when apraxics pantomime or imitate actions (Negri et al. 2007; Goldenberg and Spatt 2009; Garcea et al. 2013; Mengotti et al. 2013; for review, see Leiguarda and Marsden 2000; Goldenberg 2009; Buxbaum and Kalenine 2010; Rumiati et al. 2010). It is noteworthy that imitation of meaningless gesture is more strongly associated with lesions to the left inferior parietal lobule (Weiss et al. 2001; Tessari et al. 2007; Hoeren et al. 2014; for a neuroimaging meta-analysis, see Caspers et al. 2010), whereas tool use gesturing draws on additional stored representations, including visual and postural knowledge of actions that are processed in the posterior temporal lobe. For example, although spatiotemporal errors in meaningless gesture imitation are associated with parietal and premotor lesions, hand posture errors in tool use gesturing, reflecting knowledge of the nonarbitrary hand and finger configuration required for functional manipulation, are associated with lesions to the left posterior middle temporal gyrus (Buxbaum et al. 2014; see also Manuel et al. 2013; Hoeren et al. 2014).

Lesion-symptom mapping investigations provide evidence that the left posterior middle temporal gyrus supports action recognition and tool knowledge, as lesions to this region are associated with impairment when matching videos of tool use gestures to action verbs (Kalenine et al. 2010; Kemmerer et al. 2012) and impaired performance when naming tools but not animals (Brambati et al. 2006; Campanella et al. 2010). Functional magnetic resonance imaging (fMRI) findings in neurotypical adults are consistent with the available neuropsychological data, as there is increased blood oxygen level-dependent (BOLD) contrast in the left posterior middle temporal gyrus when participants gesture tool use (Johnson-Frey et al. 2005; Brandi et al. 2014; Vry et al. 2015), view images of tools (Chao et al. 1999; Beauchamp et al. 2002; Mahon et al. 2007; Garcea et al. 2016; for review, see Martin 2007; Lingnau and Downing 2015), and make judgments about actions (Kable et al. 2002, 2005; Wurm and Caramazza 2019), including tool use actions (Valyear and Culham 2010; Kleineberg et al. 2018). Furthermore, an emerging literature demonstrates that there is increased functional connectivity between the left inferior parietal lobule and left posterior middle temporal gyrus when neurotypical participants gesture the use of tools (Vingerhoets and Clauwaert 2015; Garcea et al. 2018; Hutchison and Gallivan 2018) and in resting-state fMRI (Simmons and Martin 2012). Consistent with this finding, the degree of resting-state functional connectivity reduction between the left inferior parietal lobule and left posterior middle temporal gyrus predicts the severity of tool use gesturing deficits in apraxia after left cerebrovascular accident (LCVA) (Watson et al. 2019).

The left medial fusiform gyrus, a region implicated in fMRI studies of tool processing (Chao et al. 1999; Mahon et al. 2007; Garcea and Mahon 2014), is sensitive to material and textural properties of manipulable objects (Cant and Goodale 2007; Cant et al. 2009) and is another region for which disconnection appears related to praxis capacity. Tool use gesturing deficits in apraxia are associated with reduced resting-state functional connectivity between the inferior parietal lobule and the left medial fusiform gyrus (Watson et al. 2019). Moreover, in preoperative neurosurgery participants, the degree of reduced BOLD contrast for tools in the left medial fusiform gyrus was associated with lesions in the left inferior parietal lobule (Garcea, Almeida, et al. 2019), suggesting that parietal lesions disrupt the processing of tools in functionally connected nodes in ventral temporal cortex.

Other nodes of relevance to praxis are precentral and prefrontal cortices, including left ventral premotor cortex and left inferior and middle frontal gyri. Lesions to the left inferior frontal or middle frontal gyri can result in tool gesturing deficits (Haaland et al. 2000; Goldenberg et al. 2007; see also Bohlhalter et al. 2011), and both regions exhibit increased functional connectivity to the left inferior parietal lobule when neurotypical participants gesture tool use (Garcea and Buxbaum 2019). In addition, greater BOLD contrast is observed in the left ventral premotor cortex when participants judge the appropriateness of an action deployed to an object, as this activation is driven by the number of potential actions associated with the object (Schubotz et al. 2014; for review, see Buxbaum 2017).

In sum, these findings indicate that in the course of generating a tool use action, conceptual attributes of actions (e.g., knowledge of hand posture and action function) and visual attributes of objects (e.g., object form, surface texture) provide key inputs to parietal sensory–motor systems in order to manipulate a tool skillfully and in accordance with its function. These action and object processes, by hypothesis, interface with frontal–motor regions critical for action selection and motor specification. We (and others) refer to the network of brain regions that collectively support the ability to recognize actions and use manipulable objects as the Tool Use Network (see Garcea and Mahon 2014; Buxbaum and Randerath 2018). Recent findings suggest that unique variance in apraxia severity may be captured by disruption of functional connectivity among nodes in the Tool Use Network (Watson et al. 2019). Though several two-route models have elucidated putative white matter tracts of relevance for visually guided action (for discussion, see Binkofski and Buxbaum 2013; Cloutman 2013), there is a paucity of structural connectivity research in apraxia. Thus, the goal of this study was to investigate the extent to which structural disconnection among nodes of the Tool Use Network is predictive of deficits in tool use gesturing.

Recent studies have raised the concern that stroke lesions invading white matter can distort the quality of diffusion data, reducing the accuracy of tractography quantification (de Groot et al. 2013; Theaud et al. 2017; Langen et al. 2018). To overcome this limitation, Greene et al. (2019) developed an analytic tool to infer structural connectivity on the basis of stroke lesion location using a large cohort of normal diffusion scans (The Human Connectome Project [HCP] database) and demonstrated that the extent of disconnection of the corticospinal tract induced by stroke lesions could reliably predict reduced contralesional grip strength scores. In the current project, we use Greene and colleagues’ structural connectivity measure to investigate structural disconnection associated with deficits in tool use gesturing.

Fifty-seven LCVA participants took part in neuropsychological testing of tool use gesturing and meaningless gesture imitation using the ipsilesional hand and underwent high-resolution structural neuroimaging. We then used support vector machines in combination with voxel-based and connectome-based lesion-symptom mapping to investigate the lesion sites and disconnection patterns, respectively, associated with reduced tool use gesturing performance, controlling for performance in meaningless gesture imitation. We reasoned that removing variance in tool use gesturing shared with meaningless gesture imitation will, by hypothesis, permit a test of structural disconnection associated with impaired retrieval of stored knowledge of tools and their associated actions, which would not be present in the context of imitating a novel, meaningless gesture. We hypothesized that disconnection of the left inferior parietal from the left middle temporal gyrus, left medial fusiform gyrus, and left precentral and prefrontal nodes would be associated with worse tool use gesturing performance.

In a second aim, we investigated lesion sites and disconnection patterns associated with motor system disconnection, as 41 of the 57 participants also took part in a test of grip strength using the ipsilesional and contralesional hand. We reasoned that the hypothesized pattern of disconnection among nodes in the Tool Use Network should not predict weakened contralesional grip strength. By contrast, we hypothesized that disconnection among pre- and postcentral gyri, and subcortical structures in the basal ganglia would be predictive of reduced grip strength of the contralesional hand after LCVA.

## Materials and Methods

### Participants

**Figure 1:**
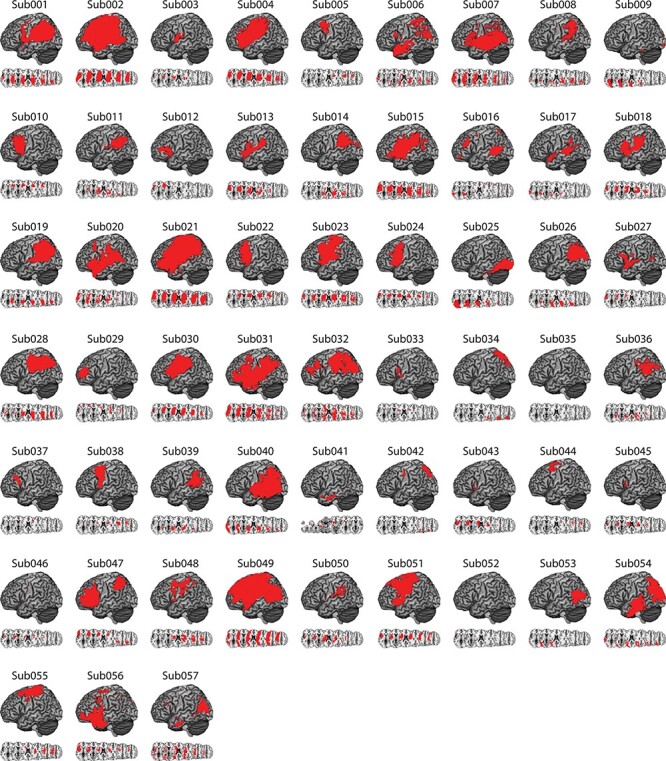
Distribution of cortical and subcortical damage in each LCVA participant. A depiction of the 57 LCVA participants’ lesions is presented on the ch2bet template. Lesions are projected on the cortical surface but include subcortical voxels using a 12-voxel search depth. Axial slices begin at the Montreal Neurological Institute (MNI) coordinate [0,0,0] and increase in 10-mm increments superiorly. Note that Sub041’s lesion rendering includes voxels below the origin.

Sixty-six chronic LCVA participants were recruited from the Neuro-Cognitive Research Registry at Moss Rehabilitation Research Institute. Of those participants, 58 completed the gesturing tool use task and the meaningless imitation task. One of the 58 participants was determined to be an outlier, and thus, all final analyses included 57 participants (28 female; mean age = 56 years, standard deviation [SD] = 11.5 years, range = 31–80 years; mean education = 14.2 years, SD = 2.8 years, range = 9–21 years). All participants were right-hand dominant (1 reported as ambidextrous) and had suffered a single left-hemisphere stroke at least 3 months prior to testing (mean number of months since stroke = 40.5 months, SD = 48.4 months; range = 4–184 months). Participants were excluded if they had a history of psychosis, drug or alcohol abuse, comorbid neurological disorder, or severe language comprehension deficits established with the Western Aphasia Battery (Kertesz 1982). See Table 1 for demographic information and Figure 1 for cortical and subcortical lesion distribution for each LCVA participant.

**Table 1:** Demographic information and lesion volume for each LCVA participant

LCVA participant	GTS HP (%)	IMI HP (%)	LH grip strength	RH grip strength	Months post stroke onset	Lesion volume (1 mm^3^)	Years of education	Gender	Age
1	0.60	0.70	22.34	20.34	174	115 118	16	F	54
2	0.75	0.50	35	_	172	258 736	18	M	68
3	1.00	0.90	_	_	7	16 978	12	M	31
4	0.83	0.90	37.67	27	112	166 393	13	M	57
5	0.63	0.60	14.67	15	7	20 190	12	F	69
6	0.73	0.70	30	20	14	76 301	18	M	64
7	0.55	0.00	27.34	_	22	171 128	18	M	51
8	0.84	0.90	30.67	33	89	51 780	21	M	79
9	0.80	0.60	20.67	16	47	82 964	14	M	53
10	0.60	0.70	15	14	4	33 183	14	F	59
11	0.93	0.70	17	13	73	37 091	14	F	55
12	0.54	0.28	13.34	15.34	114	18 528	12	F	80
13	0.83	0.50	26.34	_	84	80 020	13	F	47
14	0.88	0.70	47.67	37.34	24	47 442	12	M	53
15	0.62	0.60	39.67	_	31	179 606	15	M	57
16	0.68	0.70	31	33	6	23 141	12	M	55
17	1.00	0.60	37.67	31.67	53	20 105	21	M	56
18	0.79	0.70	17.67	_	8	71 022	16	F	60
19	0.56	0.40	14.67	22	9	62 204	18	F	32
20	0.93	0.50	24.34	29.67	68	94 536	12	F	45
21	0.24	0.50	8	_	11	303 310	12	F	65
22	0.50	0.40	23.34	28	7	61 198	12	F	71
23	0.66	0.70	28.34	_	32	136 576	12	F	46
24	0.75	0.90	33.34	26	24	52 416	14	M	48
25	0.83	0.70	18	22	9	128 897	12	F	54
26	0.76	0.90	35.67	34	7	51 399	18	M	62
27	0.76	0.80	_	_	7	20 790	12	F	50
28	0.85	0.70	41	_	10	88 046	13	M	31
29	0.90	0.70	33.34	4.34	65	27 840	12	F	39
30	0.83	0.78	29	16	6	92 744	12	M	48
31	0.70	0.70	_	_	31	200 079	12	M	61
32	0.63	0.50	26.67	8.67	19	117 809	12	F	41
33	0.79	0.40	22	24.67	40	5953	16	M	64
34	0.70	0.30	22.67	22.67	9	32 684	13	F	64
35	0.95	0.40	46.34	54	32	337	16	M	55
36	0.55	0.90	28	30.34	31	32 003	18	M	71
37	0.95	0.60	36	14.34	23	4095	18	M	63
38	0.87	0.70	42.34	14	10	27 004	12	M	48
39	0.76	0.90	26.34	34	20	29 052	12	M	58
40	0.87	0.40	31.34	35.67	17	102 522	16	F	38
41	0.90	0.70	34.34	22.34	16	14 660	18	M	53
42	0.80	0.70	21.67	20	4	9489	12	F	68
43	0.85	0.40	35.67	0	63	44 493	18	F	46
44	0.89	0.90	19.67	17.67	17	16 977	9	M	50
45	0.89	0.80	23.67	17.34	6	30 414	12	F	68
46	0.65	0.60	_	_	7	16 186	14	F	70
47	0.98	1.00	46	53.34	6	64 375	12	M	45
48	0.74	0.60	26.67	0	9	56 281	9	F	66
49	0.39	0.40	29.34	_	12	225 021	16	M	64
50	0.88	0.78	26	7.67	71	48 459	13	F	53
51	0.90	0.86	_	_	17	93 628	16	F	37
52	0.82	0.90	21.67	27.67	12	1869	12	F	67
53	0.98	0.80	36.67	32.34	12	17 706	12	M	51
54	0.89	0.71	37.34	33.34	151	80 532	16	F	55
55	0.85	0.78	30.67	_	184	62 530	13	F	60
56	0.87	0.64	23.67	10	30	87 120	18	M	76
57	0.80	0.50	45.34	_	161	96 196	13	M	60

Note: Underscores indicate participants for whom measures of right- and left-hand grip strength were not available. GTS, gesturing tool use to the sight of objects; IMI, meaningless imitation; HP, hand posture accuracy; LH, left hand; RH, right hand.

In compliance with the guidelines of the Institutional Review Board of Einstein Healthcare Network, all participants gave informed consent and were compensated for travel expenses and participation. The informed consents obtained did not include permission to make data publicly available. Accordingly, the conditions of our ethical approval do not permit anonymized study data to be publicly archived. To obtain access to the data, individuals should contact the corresponding author. Requests for data are assessed and approved by the Institutional Review Board of the Albert Einstein Healthcare Network.

### Neuropsychological Testing of Tool Use Gesturing and Meaningless Imitation

#### Gesturing Tool Use to the Sight of Objects

The gesturing tool use to the sight of objects test included 40 photographs of manipulable objects (tools) taken from the BOSS database (Brodeur et al. 2010). Tools included items with distinct use actions, including construction tools (e.g., wrench), household articles (e.g., teapot), office supplies (e.g., scissors), and bathroom items (e.g., razor). Each trial of the test began with the presentation of a 400-by-400 pixel color photograph of a tool on a computer monitor. Participants were asked to “show how you would use the tool as if you were holding and using it” with the left hand. Four practice trials with feedback (using items different than in the task itself) were given at the start of the task. As per Rothi et al. (1991), if a participant gestured the action as if their hand was the tool (body-part-as-object error), they were reminded to “show how you would use the tool as if you were actually holding it in your hand.” The first of these errors was corrected and the participant was permitted a second try (for precedent, see Tarhan et al. 2015; Watson and Buxbaum 2015; Garcea, Stoll, et al. 2019).

#### Test of Meaningless Imitation

Participants were presented with videos of an experimenter performing 10 novel gestures and were instructed to imitate the gesture. Gestures were presented twice on each trial; during the first presentation, participants were instructed to watch the gesture in its entirety; at the beginning of the second presentation, a sound was presented cueing participants to begin gesturing. The 10 novel gestures were developed to maintain similar motor characteristics of tool use gestures (e.g., plane of movement; joints moved; hand posture) but were designed such that the movement was meaningless (e.g., see Buxbaum et al. 2014). Seven of the 57 individuals took part in a version of the test with 14 meaningless actions to imitate, 10 of which were identical to the 10 meaningless actions that the remaining 50 participants gestured.

#### Coding of Action Data

Gesturing tool use and meaningless imitation tests were recorded with a digital camera and scored offline by two trained, reliable coders (Cohen’s Kappa score = 94%) who also demonstrated inter-rater reliability with previous coders in the Buxbaum lab (Cohen’s Kappa >85%; see e.g., Buxbaum et al. 2005). Both tests were coded using a portion of the detailed praxis scoring guidelines used in our previous work (see Buxbaum et al. 2000, 2005; Watson and Buxbaum 2015). In the gesturing tool use test, each gesture was given credit for semantic content unless a participant performed a recognizable gesture appropriate for a semantically related tool. Only gestures that were given credit for semantic content were scored on other action dimensions (e.g., spatiotemporal hand posture errors). Across tests of tool use gesturing and meaningless imitation, hand posture errors were assigned if the shape or movement trajectory of the hand and/or wrist was flagrantly incorrect, or if the hand or finger was used as part of the tool (i.e., body-part-as-object error, Buxbaum et al. 2005; Watson and Buxbaum 2015). This allowed us to investigate hand posture errors in the context of producing meaningful and meaningless actions and to isolate unique variance when generating errors in meaningful gesture production to test hypotheses of Tool Use Network disconnection in association with reduced performance in tool use gesturing.

Following action coding, we computed the average proportion of hand posture errors from the tool use gesturing test and from the meaningless imitation test. Consistent with past findings in our lab (Buxbaum et al. 2014), there was a significant relation between proportion of errors in tool use gesturing and meaningless imitation (*r*(55) = 0.41, *P* < 0.01). Thus, we regressed performance from the gesturing tool use test on the meaningless imitation test to obtain a residual hand posture error score; negative-going residual scores indicate worse performance when gesturing tool use relative to meaningless imitation, which was the focus of the current investigation. Residual scores were entered as the principal-dependent variable in a support vector regression voxel-based lesion-symptom mapping (SVR-VLSM) analysis. In our support vector regression connectome-based lesion-symptom mapping (SVR-CLSM) analysis, we entered residual scores derived from a model that regressed out meaningless imitation performance and total lesion volume in order to remove the influence of lesion size on tool use gesturing performance.

#### Test of Grip Strength

Forty-one of the 57 participants took part in a test of grip strength. On each trial, participants squeezed a hydraulic hand dynamometer as hard as possible using their contralesional (right) and ipsilesional (left) hands (for product details, see https://www.3bscientific.com/product-manual/W50175.pdf). Participants did not use their right hand if they had excessive weakness or poor control of the fingers or hand (likely reflecting the presence of post-stroke hemiparesis); for this reason, we did not obtain grip strength in 16 individuals. Participants took part in three trials to ensure an accurate recording of grip strength; we then averaged across the three trials to obtain a final right- and left-hand grip strength score. There was a significant correlation between right- and left-hand grip strength scores (*r*(39) = 0.48, *P* < 0.01). Thus, we regressed right-hand grip strength on left-hand grip strength to obtain a residual right-hand grip strength score; negative-going residual scores indicate reduced right-hand grip strength relative to left-hand grip strength, which was the focus of the current investigation. Residual scores were then entered as the principal-dependent variable in a SVR-VLSM analysis. In our SVR-CLSM analysis, we entered residual scores derived from a model that regressed out left-hand grip strength and total lesion volume in order to remove the influence of lesion size on grip strength.

### Neuroimaging Acquisition

#### Acquisition of Anatomic Scans

MRI scans included whole-brain T1-weighted MR images collected on a 3T (Siemens Trio, Erlangen, Germany; repetition time = 1620 ms, echo time = 3.87 ms, field of view = 192 × 256 mm, 1 × 1 × 1 mm voxels) or a 1.5T (Siemens Sonata, repetition time = 3000 ms, echo time = 3.54 ms, field of view = 24 cm, 1.25 × 1.25 × 1.25 mm voxels) scanner, using an 8- or 64-channel head coil. Lesions were manually segmented on each LCVA participant’s high-resolution T1-weighted structural images. Lesioned voxels, consisting of both gray and white matter, were assigned a value of 1 and preserved voxels were assigned a value of 0. Binarized lesion masks were then registered to a standard template (Montreal Neurological Institute “Colin27”) using a symmetric diffeomorphic registration algorithm (Avants et al. 2008, www.picsl.upenn.edu/ANTS). Volumes were first registered to an intermediate template composed of healthy brain images acquired on the same scanner. Volumes were then mapped onto the “Colin27” template to complete the transformation into standardized space. To ensure accuracy during the transformation process, lesion maps were subsequently inspected by a neurologist (H.B. Coslett), who was naïve to the behavioral data of the study. For increased accuracy, the pitch of the template was rotated to approximate the slice plane of each LCVA participant’s scan. This method has been demonstrated to achieve high intra- and inter-rater reliability (e.g., see Schnur et al. 2009). See Figure 1 for a rendering of each LCVA participant’s lesion.

### Support Vector Regression Lesion-Symptom Mapping Analyses

#### SVR-VLSM Analyses

SVR-VLSM was performed in MATLAB 2017B using a toolbox developed by DeMarco and Turkeltaub (2018) (https://github.com/atdemarco/svrlsmgui/). SVR-VLSM is a multivariate technique that uses machine learning to determine the association between lesioned voxels and behavior when considering the lesion status of all voxels submitted to the analysis. It overcomes several limitations of univariate VLSM, including inflated false positives from correlated neighboring voxels (Pustina et al. 2018), type II error due to correction for multiple comparisons (Bennett et al. 2009), and uneven statistical power due to biased lesion frequency as a function of vascular anatomy (Mah et al. 2014; Sperber and Karnath 2017). SVR-VLSM has been shown to be superior to VLSM when multiple brain areas are involved in a single behavior (Mah et al. 2014; Herbet et al. 2015; Mirman, Zhang, et al. 2015b; but see Sperber, Wiesen, and Karnath 2019b; for discussion, see Zhang et al. 2014).

We performed two SVR-VLSM analyses. The first analysis tested the hypothesis that impaired tool use gesturing, controlling for variability in meaningless gesture imitation, would be associated with lesions to the left inferior parietal lobule, the left middle temporal gyrus, and left inferior and middle frontal gyri. The dependent measure was hand posture accuracy scores when gesturing tool use, residualized against hand posture accuracy scores when imitating meaningless gestures. A second SVR-VLSM analysis tested the hypothesis that reduced right-hand grip strength would be associated with lesions to the left primary motor cortex, premotor cortex, and subcortical structures including the basal ganglia. The dependent measure was right-hand grip strength score residualized against left-hand grip strength scores.

Only voxels lesioned in at least 10% of participants (5 participants in the analysis of tool use gesturing; 4 participants in the analysis of right-hand grip strength) were included. We controlled for variability in lesion volume using the “Direct Total Lesion Volume Control” corrective method (see DeMarco and Turkeltaub 2018). Five-fold cross-validation was implemented, in which 80% of the participants’ lesions and behavioral data were used to train a classifier and the remaining 20% of participants’ lesions and behavioral data were used to test the classifier. This procedure was iterated 5 times to ensure that each unique subset of participant data was independently used for training and testing, and the resulting 5 maps of beta values were averaged together to derive a final averaged map of voxelwise beta values. Voxelwise statistical significance was then determined using a Monte Carlo style permutation analysis in which the behavioral data were randomly assigned to a lesion map, and the same procedure as described above was iterated 10 000 times. Voxelwise *z*-scores were then computed for the true data in relation to the mean and SD of voxelwise null distributions; the resulting *z*-score map was set to a threshold of *z* < −1.65 (*P* < 0.05, one-tailed) to determine chance-level likelihood of a lesion-symptom relation. We then further restricted the resulting map by eliminating any clusters with fewer than 500 contiguous voxels (see Lacey et al. 2017; Skipper-Kallal et al. 2017; Garcea, Stoll, et al. 2019). The Anatomical Automatic Labeling atlas (Tzourio-Mazoyer et al. 2002) was used to assess overlap of significant voxels in the SVR-VLSM analyses with cortical and subcortical regions.

##### Support Vector Regression Connectome-Based Lesion-Symptom Mapping

We used support vector regression in tandem with structural connectivity to investigate disconnection among left-hemisphere cortical nodes in relation to tool use gesturing performance (e.g., see Yourganov et al. 2016; Bonilha et al. 2017; Gleichgerrcht et al. 2017; Pustina et al. 2018). We performed two SVR-CLSM analyses. The first analysis tested the hypothesis that impaired tool use gesturing would be associated with disconnection of the left inferior parietal from the left middle temporal gyrus, left medial fusiform gyrus, and left precentral and prefrontal nodes. A second SVR-CLSM analysis tested the hypothesis that reduced right-hand grip strength would be associated with disconnection among pre- and postcentral gyri and subcortical structures in the basal ganglia.

First, each LCVA participant’s stroke lesion was drawn on their corresponding native T1-weighted magnetization prepared rapid gradient echo (MP-RAGE) volume and then normalized to a custom T1-weighted template constructed from 40 participants of the HCP (Greene et al. 2018) using cost function masking in ANTS (Avants et al. 2008). We then parcellated each participant’s MP-RAGE volume using the Lausanne 2008 atlas (Hagmann et al. 2008; Daducci et al. 2012). The Lausanne atlas contains 130 nodes distributed throughout the right and left hemispheres; for the purpose of the current investigation, we analyzed the 64 left-hemisphere nodes only, removing the left cerebellum (see Supplementary Table 1 for mean Montreal Neurological Institute coordinates of all left hemisphere nodes).

Structural connectivity between a given node pair was estimated for each of 210 neurotypical participants’ shortest path tractography derived from the diffusion scans of the HCP dataset. Then, a given LCVA participant’s lesion was projected into these scans and the impact of the lesion on the shortest path tractography was estimated. Specifically, the percent loss in structural connectivity between any two nodes was calculated as the proportion of the shortest path tractography in the HCP dataset intersecting the lesion relative to the total shortest path probability shared between any given node pair in the HCP dataset (for methodological detail, see Greene et al. 2019). The result is a 130 × 130 disconnectome map, representing node-to-node (hereafter, edges) structural disconnection inferred on the basis of stroke lesion location for each participant. Disconnection values were continuous, ranging from 1 (complete disconnection) to 0 (no disconnection). Edges were included in group-level analyses only if they were lesioned in at least 10% of participants (5 participants in the analysis of tool use gesturing; 4 participants in the analysis of right-hand grip strength), akin to the SVR-VLSM approach.

Prior to conducting the SVR-CLSM analysis, we regressed variability in edge-level disconnection on variability of three factors: 1) cumulative disconnection of the regions identified in the SVR-VLSM analysis; 2) total lesion volume of each LCVA participant; and 3) the number of months post-stroke of each LCVA participant.^1^ In the analysis of tool use gesturing, cumulative structural disconnection was calculated for the left frontal cortex (inferior, middle, and superior frontal gyri), the left temporal lobe (superior temporal gyrus, middle temporal gyrus, transverse temporal, bank of the superior temporal sulcus), and the left parietal lobe (supramarginal gyrus, angular gyrus, superior parietal lobule). In the analysis of right-hand grip strength, cumulative structural disconnection was calculated for left cortical (pre- and postcentral gyri) and subcortical areas (left insula, and left subcortical regions including the thalamus, caudate, putamen, pallidum, and nucleus accumbens). In this way, we use the SVR-VLSM results as a “localizer” map to independently identify regions of relevance in the SVR-CLSM analysis, and by regressing cumulative structural disconnection of those sites out of edge-level disconnection variability, we ensure that our observed disconnection patterns exist over and above cumulative disconnection of a given node contributing to the behavioral task of relevance.

The residual disconnection values were then entered in a support vector regression analysis (“fitrsvm” function in MATLAB 2017B) using a Gaussian kernel function (“rbf”) with a kernel scale value set to 1. Cross-validation was used, in which 80% of the disconnection data were used to train the model to learn the relation between disconnection and behavioral scores and 20% of the disconnection data were used to test the robustness of that model using a left-out sample. After iterating this procedure 5 times, all data were used to independently train and test the classifier, which resulted in 5 disconnectome maps of feature weights (beta values), indicating the strength of a given edge predicting behavioral scores. We then averaged across the 5 disconnectome maps to obtain a final map (64 × 64 matrix of left-hemisphere disconnection).

To interpret the strength of edge-level beta weights, we conducted a Monte Carlo style permutation analysis in which we randomly assigned behavioral scores to disconnectome maps and repeated the analysis 10 000 times; all other aspects of the permutation analysis were identical to the analysis of the true data. The result of the permutation analysis is a distribution of feature weights for a given edge that arise due to chance; we then *z*-score the feature weights of the true data relative to the mean and SD of the null distribution. The resulting *z*-value matrix was set to a threshold of *z* = −1.65 (*P* < 0.05, one-tailed) to identify above-chance feature weight values.

#### Calculating Maximally Disconnected Subgraphs

Because the resulting disconnectome can be quite extensive with many region pairs having negligible connectivity loss (rendering visualization and interpretation challenging), we reduce the dimensionality of the *z*-score disconnectome by extracting a maximally disconnected subgraph. The maximally disconnected subgraph contains regions and edges with the greatest shared disconnection associated with reduced performance on the test of interest (for precedent in using this subgraph analysis over stroke disconnection data, see Greene et al. 2019). Critically, only edges that survive Monte Carlo permutation analysis are entered into the maximally disconnected subgraph analysis.

## Results

### SVR-VLSM Results

**Figure 2:**
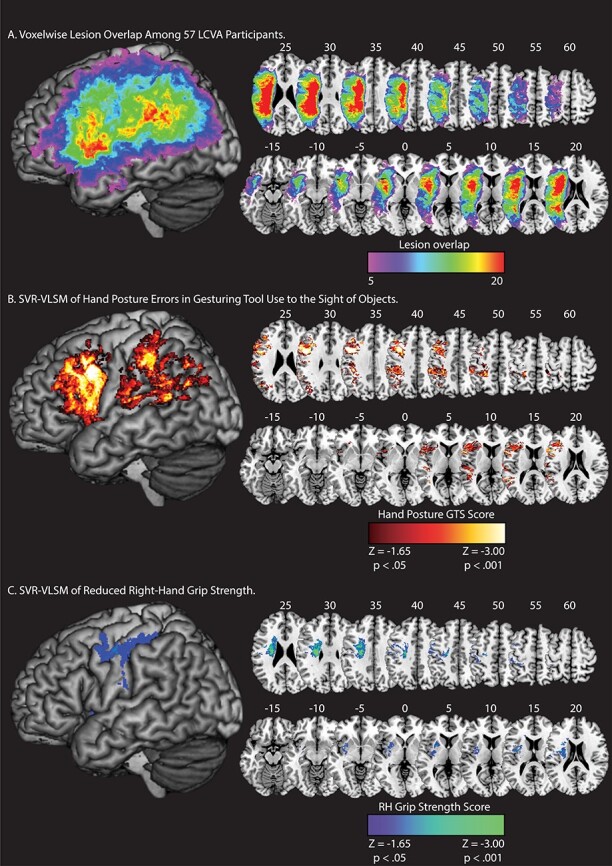
Lesion overlap and SVR-VLSM results. (*A*) Voxelwise lesion overlap among 57 participants. Only voxels with at least 5 lesions are included in the analysis of Tool Use Gesturing. (*B*) Voxels associated with reduced performance in tool use gesturing (greater hand posture errors in tool use gesturing controlling for hand posture errors in meaningless imitation; red-to-white scale). (*C*) Voxels associated with reduced right-hand grip strength (weaker grip strength with the right hand controlling for grip strength of the left hand; blue-to-green scale). Whole-brain results are rendered in MNI space in 5-mm increments. SVR-VLSM maps are set to a voxelwise threshold of *P* < 0.05 with 10 000 iteration Monte Carlo style permutation analysis; clusters are included if they are greater than or equal to 500 contiguous 1 mm^3^ voxels.

Figure 2*A* depicts lesion overlap among the 57 participants with high-resolution MRI anatomical data (see Supplementary Fig. 1 for a rendering of structural disconnection overlap among the 57 participants). Two SVR-VLSM analyses were conducted; in the first analysis, we identified voxels in which lesions were associated with worse performance when gesturing tool use to the sight of objects. In the second analysis, we identified voxels in which lesions were associated with reduced right-hand grip strength.

#### Tool Use Gesturing

Lesions to two large clusters were associated with reduced tool use gesturing performance (Fig. 2*B*). The first cluster included the left inferior parietal lobule (left supramarginal gyrus, left angular gyrus), the left superior parietal lobule, the left postcentral gyrus, the left superior temporal gyrus, the left middle temporal gyrus, and posterior voxels including portions of middle and superior occipital gyri (see Table 2A). A second cluster included voxels in the left inferior frontal gyrus (pars opercularis, pars triangularis), the middle frontal gyrus, and the superior frontal gyrus. This cluster extended posteriorly to include the left precentral gyrus and inferiorly to include the insula and putamen (see Table 2A). These findings replicate our prior univariate work (Buxbaum et al. 2014; Watson and Buxbaum 2015) using a multivariate approach (see Supplementary Fig. 2).

**Table 2:** Peak MNI coordinates identified in the SVR-VLSM analysis of errors in tool use gesturing, controlling for errors in meaningless imitation (A), and reduced right-hand grip strength, controlling for a reduction in left-hand grip strength (B)

AAL label	AAL number	Mean center of mass (XYZ)			Peak *z*-score	Number of voxels in cluster
A. Errors in tool use gesturing, controlling for errors in meaningless imitation						
Precentral gyrus	1	−45	8	35	−3.72	6020
Superior frontal gyrus	3	−20	22	44	−2.32	7
Middle frontal gyrus	7	−36	11	39	−3.72	3726
Pars opercularis	11	−42	7	29	−3.72	3971
Pars triangularis	13	−52	18	30	−3.43	5172
Pars orbitalis	15	−28	29	−8	−2.30	3
Rolandic operculum	17	−52	6	9	−3.19	393
Insula	29	−26	25	15	−2.91	1465
Superior occipital gyrus	49	−25	−66	25	−2.11	7
Middle occipital gyrus	51	−36	−69	28	−2.83	599
Postcentral gyrus	57	−31	−33	45	−3.24	3178
Superior parietal lobule	59	−32	−60	45	−2.37	44
Inferior parietal lobule	61	−41	−29	38	−3.29	4923
Supramarginal gyrus	63	−63	−43	25	−3.09	1214
Angular gyrus	65	−42	−64	40	−3.12	1449
Putamen	73	−26	9	16	−2.03	5
Heschl’s gyrus	79	−42	−25	11	−2.64	130
Superior temporal gyrus	81	−52	−32	13	−3.43	2149
Superior temporal pole	83	−59	8	1	−2.47	21
Middle temporal gyrus	85	−48	−52	13	−3.72	1259
B. Weakened right-hand grip strength, controlling for left-hand grip strength						
Precentral gyrus	1	−41	−3	27	−2.89	746
Middle frontal gyrus	7	−32	14	37	−2.21	17
Pars opercularis	11	−38	7	17	−1.98	4
Rolandic operculum	17	−39	−6	21	−2.7	545
Insula	29	−35	−2	16	−2.83	1055
Amygdala	41	−29	−6	−10	−1.81	3
Postcentral gyrus	57	−39	−17	42	−2.56	2214
Superior parietal lobule	59	−35	−40	57	−1.67	2
Inferior parietal lobule	61	−45	−25	41	−1.70	20
Caudate	71	−19	12	24	−2.74	241
Putamen	73	−29	−2	13	−2.74	2067
Pallidum	75	−21	2	7	−2.03	29
Superior temporal gyrus	81	−43	−10	1	−1.90	1

Note: Region labels were derived from the Automated Anatomical Labeling (AAL) template.

#### Reduced Grip Strength

Lesions to two clusters were associated with reduced right-hand grip strength (see Fig. 2*C*). The first cluster included pre- and postcentral gyri, the left inferior and superior parietal lobule, and the left middle frontal gyrus. A second cluster included medial portions of the left superior temporal gyrus, the left insula, and basal ganglia (including caudate, putamen, and pallidum), and the left amygdala (see Table 2B). These results confirm that reduced right-hand grip strength is associated with lesions to the cortical motor system and to additional regions including basal ganglia and insula, with little overlap of the lesion sites associated with reduced tool use gesturing performance.

### SVR-CLSM Results

#### Tool Use Gesturing Maximally Disconnected Subgraph

**Figure 3:**
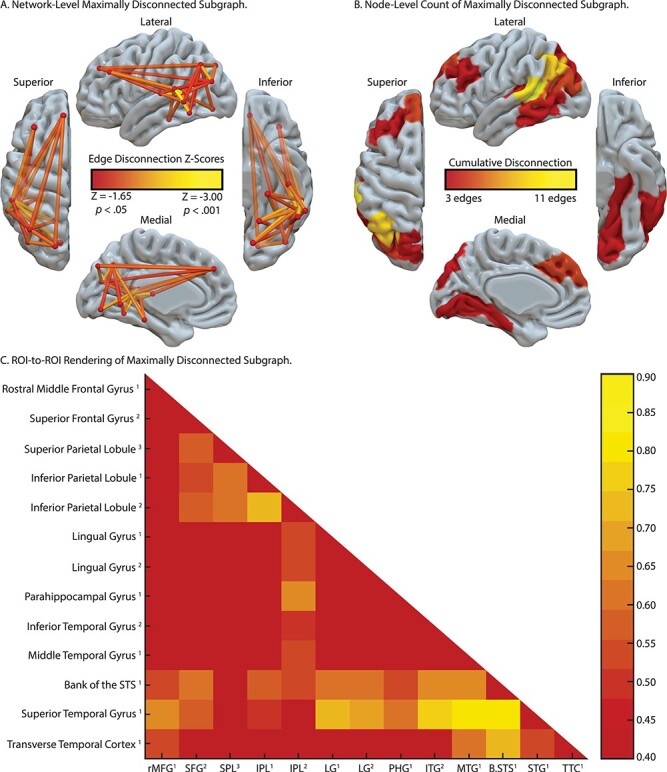
SVR-CLSM of hand posture errors in tool use gesturing, controlling for imitation of meaningless gestures and total lesion volume. (*A*) Maximally disconnected edges associated with reduced performance in tool use gesturing (greater hand posture errors in tool use gesturing controlling for hand posture errors in meaningless imitation and total lesion volume; red-to-yellow scale). (*B*) Cumulative node-level disconnection associated with reduced tool use gesturing projected on the cortical surface. (*C*) A heatmap of the disconnected subgraph depicting ROI-to-ROI disconnection patterns projected in Figure 3*A*.

As shown in Figure 3*A*, there were four clusters identified as maximally disconnected in association with reduced tool use gesturing performance, controlling for imitation of meaningless gestures and total lesion volume. First, we identified inferior-going disconnection between the left inferior parietal lobule and superior temporal sulcus, transverse temporal cortex, middle temporal gyrus, inferior temporal gyrus, lingual gyrus, and parahippocampal gyrus. Second, we observed disconnection among inferior parietal lobule subregions and the superior parietal lobule. Third, the superior frontal gyrus was associated with disconnection to inferior and superior parietal regions. Fourth, the rostral middle frontal gyrus was associated with disconnection to the superior temporal gyrus, transverse temporal cortex, and bank of the superior temporal sulcus. (see Fig. 3*A*, and Table 3). Aggregating node-level disconnection resulted in a map of cumulative disconnection count, which identifies the left inferior parietal lobule, superior temporal gyrus, and bank of the superior temporal sulcus as the most disconnected nodes associated with reduced tool use gesturing performance (see Fig. 3*B* and Table 3). Edge-level disconnection values are reported as a heatmap in Fig. 3*C*. For a rendering of the full *z*-score matrix, see Supplementary Fig. 3.

#### Reduced RH Grip Strength Maximally Disconnected Subgraph

**Figure 4:**
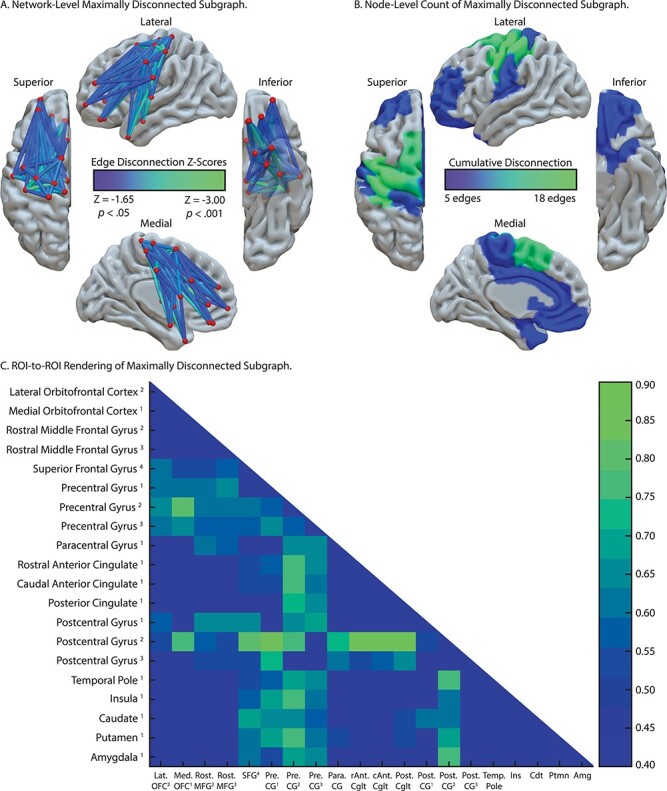
SVR-CLSM of reduced right-hand grip strength, controlling for left-hand grip strength and total lesion volume. (*A*) Maximally disconnected edges associated with reduced right-hand grip strength (weak grip strength with the right hand controlling for grip strength of the left hand and total lesion volume; blue-to-green scale). (*B*) Cumulative node-level disconnection associated with reduced right-hand grip strength projected on the cortical surface. (*C*) A heatmap of the disconnected subgraph depicting ROI-to-ROI disconnection patterns projected in Figure 4*A*.

As shown in Fig. 4*A*, pre- and postcentral gyri and the superior frontal gyrus were identified as maximally disconnected in association with reduced right-hand grip strength, controlling for left-hand grip strength and total lesion volume. These nodes exhibited disconnection to three distributed clusters. The first cluster included lateral and medial orbitofrontal cortices and the rostral middle frontal gyrus. The second cluster included posterior and anterior cingulate cortices. The third cluster included the insula, caudate, putamen, and amygdala, and extended laterally and anteriorly to include the temporal pole. Two additional clusters included disconnection between posterior cingulate cortex, caudate, and putamen and between paracentral gyrus, rostral middle frontal gyrus, and putamen (see Table 4). Consistent with the edge-based findings, the aggregate node-level disconnection count identifies pre- and postcentral gyri and medial superior frontal gyrus as maximally disconnected in association with reduced right-hand grip strength (see Fig. 4*B* and Table 4). Edge-level disconnection values are reported as a heatmap in Fig. 4*C*. For a rendering of the full *z*-score matrix, see Supplementary Fig. 4.

**Table 3:** Maximally disconnected subgraph of hand posture errors when gesturing tool use, controlling for imitation of meaningless gestures and total lesion volume

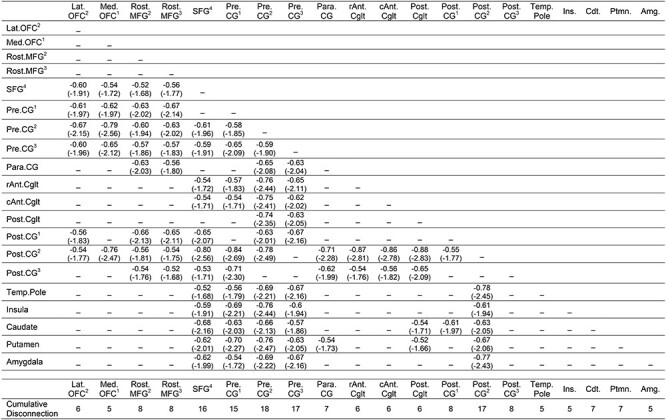

Note: Feature weights (range: max, −0.50; min, −0.82) and *z*-scores (in parentheses) are listed. rMFG, rostral middle frontal gyrus; SFG, superior frontal gyrus; SPL, superior parietal lobule; LG, lingual gyrus; PHG, parahippocampal gyrus; ITG, inferior temporal gyrus; MTG, middle temporal gyrus; B.STS, bank of the superior temporal sulcus; STG, superior temporal gyrus; TTC, transverse temporal cortex. Numbers in superscript indicate anatomical subregions from the Lausanne atlas.

**Table 4:** Maximally disconnected subgraph of reduced right-hand grip strength, controlling for left-hand grip strength and total lesion volume

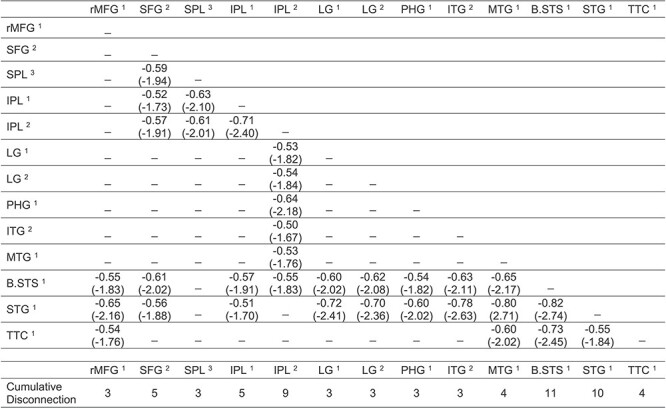

Note: Feature weights (range: max, −0.52; min −0.87) and *z*-scores (in parentheses) are listed. Lat. OFC, lateral orbitofrontal cortex; Med. OFC, medial orbitofrontal cortex; Pre. CG, precentral gyrus; Para. CG, paracentral gyrus; rAnt. Cglt, rostral anterior cingulate; cAnt. Cglt, caudal anterior cingulate; Post. Cglt, posterior cingulate; Post. CG, postcentral gyrus; Temp. pole, temporal pole. Numbers in superscript indicate anatomical subregions from the Lausanne atlas.

### Post hoc Analysis of Left Inferior Parietal Lobule Disconnection

Prior work has identified reduced functional connectivity between the left inferior parietal lobule and left medial fusiform gyrus in association with the severity of tool use gesturing. Although parietal-to-fusiform disconnection was significantly associated with hand posture errors in tool use gesturing (see Supplementary Fig. 3), it was not identified in the maximally disconnected subgraph analysis. We therefore conducted a post hoc analysis using linear regression to investigate left inferior parietal disconnectivity. The procedure was carried out in three steps. First, as described above, we used residual disconnection values after removing variability in disconnection associated with cumulative disconnection from nodes identified in the SVR-VLSM analysis, total lesion volume, and months post onset. We then computed the correlation between disconnection of the left inferior parietal lobule (IPL^2^ identified in the maximally disconnected subgraph analysis in Figure 3) with all other left hemisphere nodes and imposed three criteria to determine the significance of disconnection in association with severity of tool use gesturing performance.

First, we inspected connectivity patterns only if the node under consideration was outside of the lesion territory (see Fig. 2*A* for lesion overlap map). We did this by identifying candidate nodes with less than 1% overlap between the voxels in the remote node and the lesion overlap map. This first criterion ensures that parietal disconnection to remote nodes will not be driven by weak signal at the remote site. Second, the connectivity relation (correlation value) with the left inferior parietal node was considered if it was significant (minimum *r* < −0.22, *P* > 0.05, one-tailed). Third, we conducted a Monte Carlo style permutation analysis in which we randomly assigned the behavioral data to the disconnection data using 10 000 iterations to derive a null distribution from which to *z*-score the true data. Thus, all disconnection to the inferior parietal node 1) needed to exist outside of the lesion territory, 2) needed to predict a significant amount of variance in tool use gesturing performance, and 3) needed to be at least 2 SD (Z < −1.96) outside the mean of a null distribution for a given edge as determined by Monte Carlo style permutation analysis.

**Figure 5:**
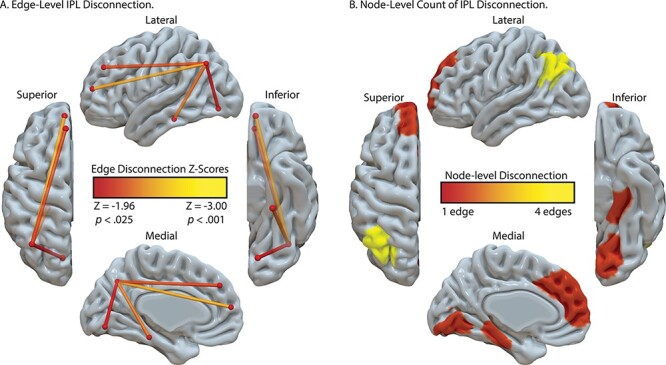
Analysis of IPL disconnection associated with hand posture errors in tool use gesturing, controlling for imitation of meaningless gestures and total lesion volume. (*A*) Edge-level disconnection identifies the left ventral temporal cortex and medial frontal regions as sites that exhibit a disconnection to the left inferior parietal lobule. (*B*) Cumulative node-level disconnection count associated with reduced tool use gesturing performance is projected on the cortical surface.

Figure 5 depicts the result of this analysis. We found significant and robust disconnection between the left inferior parietal lobule and 1) ventral temporal regions including the lingual gyrus and parahippocampal gyrus and 2) between the inferior parietal lobule and superior frontal gyrus (see Fig. 5*A*; see Table 5 for correlation values and *z*-score values). The node-level rendering of inferior parietal disconnection depicts the full extent of ventral temporal cortex and frontal involvement (Fig. 5*B*). Note that the correlation between the left inferior parietal lobule and left fusiform gyrus disconnection was significant (*r*(55) = −0.24, *P* < 0.05) but did not survive the permutation analysis (*z* = −1.80).

**Table 5:** Analysis of left inferior parietal lobule disconnection in relation to hand posture errors in tool use gesturing, controlling for imitation of meaningless gestures and total lesion volume

Region-of-interest	Correlation value	Significance value	*z*-score
Superior frontal gyrus^1^	−0.34	0.01	−2.53^*^
Superior frontal gyrus^2^	−0.29	0.02	−2.17^*^
Isthmus cingulate cortex^1^	−0.25	0.03	−1.89
Cuneus^1^	−0.25	0.03	−1.90
Pericalcarine cortex^1^	−0.25	0.03	−1.86
Lateral occipital cortex^1^	−0.23	0.04	−1.73
Lingual gyrus^1^	−0.26	0.03	−1.96^*^
Lingual gyrus^2^	−0.25	0.03	−1.88
Fusiform gyrus^1^	−0.24	0.04	−1.80
Parahippocampal gyrus	−0.30	0.01	−2.33^*^
Hippocampus	−0.23	0.05	−1.69

Note: Asterisks denote regions that satisfy three inclusion criteria. Numbers in superscript indicate anatomical subregions from the Lausanne atlas.

## General Discussion

Prior work has identified the Tool Use Network—a whole-brain network of regions working in concert to support the retrieval of action and object knowledge in the service of implementing object-directed action. We performed SVR-VLSM and SVR-CLSM analyses with data from 57 LCVA participants to elucidate the relation among tool use gesturing performance, lesion location, and structural disconnection. To our knowledge, this is the first study to combine lesion- and connectome-based symptom mapping in tandem with support vector machine learning to investigate the local and nonlocal effects of LCVA lesions on tool use ability in apraxia. Moreover, the analysis of reduced contralesional grip strength permitted us to test the specificity of disconnection within the Tool Use Network as a key substrate that gives rise to reduced tool use gesturing, and not reduced grip strength.

Consistent with the predictions of neurocognitive models of praxis (e.g., see Binkofski and Buxbaum 2013; Buxbaum 2017), impairments in tool use gesturing were associated with lesions to the left inferior parietal lobule, the left inferior and middle frontal gyri, and the left posterior middle temporal gyrus that extended into ventral occipitotemporal cortex. These results are in agreement with prior univariate VLSM analyses, including results from our lab (Buxbaum et al. 2014; Tarhan et al. 2015; Watson and Buxbaum 2015), and other key findings in the literature (Haaland et al. 2000; Manuel et al. 2013; Martin et al. 2016; Dressing et al. 2018; Sperber, Wiesen, Goldenberg, et al. 2019a). In contrast, a reduction in right-hand grip strength, controlling for shared variance in left-hand grip strength, was associated with lesions in the left pre- and postcentral gyri, the left insula, and in voxels extending subcortically to include regions of the basal ganglia (putamen, pallidum, caudate). These findings are in accordance with prior univariate VLSM analyses of reduced contralesional grip strength (Goldenberg and Spatt 2009; Greene et al. 2019) and demonstrate that the lesion sites associated with reduced strength of the right hand minimally overlap with nodes of the Tool Use Network.

We reasoned that tool use gesturing ability would be associated with the degree of disconnection among nodes of the Tool Use Network, controlling for shared variance when imitating meaningless gestures and total lesion volume. Our SVR-CLSM analysis revealed that the degree of hand posture errors when gesturing tool use was associated with disconnection among nodes in the left inferior parietal lobule, the left superior and middle frontal gyri, the left middle temporal gyrus, and left ventral (lingual gyrus) and mesial (parahippocampal gyrus, hippocampus) temporal cortices. Post hoc analyses identified a comparable finding using a traditional linear regression approach. Voxels in the temporoparietal junction (superior temporal sulcus, inferior parietal lobule) were strongly disconnected from nearly all regions identified, indicating that damage to the white matter adjacent to temporoparietal cortex has long-range disconnective effects. This is due, in part, to the proximity of white matter pathways medial to the superior temporal gyrus, an issue we return to below.

We then used SVR-CLSM over right-hand grip strength data to test the hypothesis that reductions in contralesional grip strength would be associated with disconnection among nodes of the cortical and subcortical motor system, largely bypassing nodes of the Tool Use Network. Consistent with our hypothesis, we found reduced right-hand grip strength in association with disconnection among pre- and postcentral gyri, the superior frontal gyrus, anterior and posterior cingulate cortices, basal ganglia structures, the amygdala, and prefrontal cortex. Critically, there was minimal overlap between nodes of the Tool Use Network and regional disconnection associated with reduced right-hand grip strength.

### Implications for Neurocognitive Models of Praxis in the Human Brain

We observed that structural disconnection with the left inferior parietal lobule was associated with reduced tool use gesturing performance in apraxia. The left inferior parietal lobule forms a core component of the ventro-dorsal stream, a visuomotor processing pathway that supports the retrieval of manipulation knowledge for tool use, interfacing current visual input with conceptual representations of actions (Binkofski and Buxbaum 2013). In neurotypical adults, diffusion tractography studies have identified the requisite connectivity to interface the left inferior parietal lobule with other nodes in the Tool Processing Network. For example, posterior fibers of the arcuate fasciculus (pAF) provide one anatomic substrate to connect the lateral and posterior middle temporal gyrus with the left inferior parietal lobule (Ramayya et al. 2010; see also Barbeau et al. 2020), and structural connectivity between the left inferior parietal lobule and frontal-motor sites, subserved by fibers of the superior longitudinal fasciculus (SLF), is critical for action selection and planning (Rushworth et al. 2006; Ramayya et al. 2010; Caspers et al. 2011; Ruschel et al. 2014). Furthermore, lesions involving the inferior parietal lobule and adjacent voxels overlapping with the SLF are associated with poor tool use gesturing in apraxia (Bi et al. 2015; Watson and Buxbaum 2015; Garcea, Stoll, et al. 2019), suggesting that sensory-to-motor mapping in the action domain may be underpinned in part the SLF (for discussion of the SLF/AF and its role in other cognitive domains, see Catani and Mesulam 2008; Dick et al. 2014; Ivanova et al. 2016; Chernoff et al. 2020; Forkel et al. 2020).

In contrast, the ventral fiber pathway runs inferior to the superior temporal gyrus, coursing anteriorly and ventrally through fibers of the extreme capsule and uncinate fasciculus, and is argued to play a domain-general role in conceptual processing across numerous cognitive domains, including tool use pantomiming (Vry et al. 2015) and language (Saur et al. 2008; Weiller et al. 2011; Rauschecker 2012; Rijntjes et al. 2012). Our analyses did not identify regions connected by the ventral pathway (e.g., the left inferior frontal gyrus), suggesting that the integrity of this tract may not be related to tool use gesturing integrity in apraxia. Given prior evidence that lesions involving the white matter adjacent to the inferior frontal gyrus (uncinate fasciculus, left inferior fronto-occipital fasciculus, anterior thalamic radiations) were associated with impaired selection of verbal and nonverbal conceptual knowledge (e.g., see Mirman, Chen, et al. 2015a; see also Han et al. 2013), it remains a possibility that the ventral fiber pathway supports tool action selection. However, given that current lesion evidence suggests that action selection in tool use is mediated by frontoparietal structures via the SLF (e.g., see Watson and Buxbaum 2015; Garcea, Stoll, et al. 2019), it will be important for future VLSM and CLSM work to determine the extent to which damage to the ventral fiber pathway, controlling for damage of SLF fibers, is associated with action selection difficulties.

More recently, it has been argued that fibers of the vertical occipital fasciculus (VOF) provide a substrate to connect the ventral and dorsal object processing pathways (Yeatman et al. 2014). The VOF runs lateral to the inferior longitudinal fasciculus and posterior to the arcuate fasciculus, connecting ventral occipitotemporal cortex with the posterior and inferior parietal lobule (Weiner et al. 2017). Though the integrity of VOF fibers is implicated in visual processing of faces (Weiner et al. 2016), written text (Yeatman et al. 2013), and objects (Freud et al. 2016), Budisavljevic et al. (2018) recently demonstrated that the speed with which participants reached maximum grip aperture when grasping an object was predicted by structural integrity of the VOF. Specifically, participants’ faster opening of the hand when grasping an object was associated with faster transfer of information between visual perceptual processing (analysis of shape, form, and surface texture; ventral stream) and object-directed grasping (dorsal stream). Our post hoc analysis identified structural disconnection between the left inferior parietal lobule and ventral occipitotemporal cortex in association with reduced tool use gesturing. It therefore remains a possibility that lesion to the posterior inferior parietal lobule damages superior terminations of the VOF, which subsequently contributes to tool use gesturing impairment in apraxia.

Although this speculation needs to be evaluated in future empirical work, prior studies in neurotypical participants have demonstrated increased functional connectivity between the inferior parietal lobule and ventral occipitotemporal cortex (e.g., left medial fusiform gyrus) in tool and action processing (Assmus et al. 2007; Garcea and Mahon 2014; Stevens et al. 2015; Garcea et al. 2018), and recent lesion evidence suggests that inferior parietal-to-medial fusiform connectivity disruption predicts abnormal tool processing (Garcea, Almeida, et al. 2019) and tool use gesturing ability (Watson et al. 2019). In this context, it will be important to consider whether alternative fiber pathways, including the temporoparietal aslant tract (Panesar et al. 2019) or middle longitudinal fasciculus (Makris et al. 2017; Kalyvas et al. 2020), are tracts disconnected in association with apraxia severity.

To bring our results into register with the apraxia literature, tool use gesturing performance is associated with distributed structural connectivity among the left inferior parietal lobule and lateral and inferior temporal cortices. Voxels in lateral occipitotemporal cortex respond to images of hands (Orlov et al. 2010) and tools (Bracci et al. 2012) and exhibit functional connectivity to the left inferior parietal lobule (Mahon et al. 2007; Bracci et al. 2012; Chen et al. 2017). Furthermore, parietal disconnection extended to ventral temporal cortex to include the fusiform gyrus, consistent with prior functional connectivity findings in apraxia (Watson et al. 2019) and in neurotypical adults (Garcea and Mahon 2014; see also Stevens et al. 2015; Chen et al. 2017). In light of these findings, it has been argued that the processing of object properties in ventral temporal cortex provides an input to the praxis system, because the extraction of visual attributes of objects (e.g., whether an object is slippery, hot, or sharp) as well as stored knowledge of material properties (e.g., object weight; Gallivan et al. 2014) informs the retrieval of hand posture and deployment of an object-directed grasp when functionally manipulating a tool (Almeida et al. 2013; Mahon et al. 2013; Garcea and Mahon 2019; for discussion, see Gallivan and Culham 2015).

These action and object retrieval processes will, by hypothesis, interface with frontal-motor structures of relevance for action selection and motor implementation. We found that structural connectivity between the left inferior parietal lobule and superior frontal gyrus was strongly predictive of tool use gesturing ability. Prior fMRI work has implicated dorsolateral prefrontal regions in attentional control and response selection (for meta-analysis, see Cieslik et al. 2015), and lesions to middle and inferior frontal gyri are associated with tool use pantomiming deficits (Haaland et al. 2000; Goldenberg et al. 2007; Watson and Buxbaum 2015; see also Bohlhalter et al. 2011). Considering that several fMRI studies have reported increased BOLD contrast in the inferior frontal gyrus when gesturing tool use (e.g., see Brandi et al. 2014; Vry et al. 2015), it is surprising that the maximally disconnected subgraph analysis did not reveal increased disconnection with the left inferior frontal gyrus. Although there is a paucity of structural connectivity research in apraxia, our results are consistent with Bi et al.’s (2015) finding that the severity of tool use ability was associated with structural disconnection of dorsal and superior frontal cortices. Their superior frontal area was in close anatomical proximity to the superior frontal gyrus that we identified as disconnected with the inferior parietal lobule (see Fig. 3), suggesting that frontoparietal damage has detrimental effects upon action selection and gesture implementation (for recent evidence, see Rosenzopf et al. 2020).

### Limitations

Whereas our SVR-VLSM result is consistent with a large body of work demonstrating that apraxia is associated with lesions to the supramarginal gyrus and angular gyrus (for review, see Johnson-Frey 2004; Goldenberg 2009; Orban and Caruana 2014; Buxbaum and Randerath 2018), our SVR-CLSM analysis identified the posterior inferior parietal lobule in the vicinity of the left angular gyrus. This may be driven in part by the use of shortest path tractography, which identifies the optimal path between nodes such that the probability that adjacent voxels form a contiguous, short path between nodes is high. Visual inspection of the left inferior parietal lobule subregion confirms its anatomical proximity to posterior fibers of the SLF/AF (see Supplementary Fig. 5), indicating that lesions invading white matter adjacent to the posterior inferior parietal lobule are likely to disconnect the inferior parietal lobule from ventral and lateral temporal cortices. Importantly, prior work indicates that fibers of the VOF lie posterior to fibers of the SLF/AF (Weiner et al. 2017), which is also anatomically proximal to white matter voxels adjacent to the posterior inferior parietal lobule area identified in our analysis (e.g., see Jitsuishi et al. 2020). Thus, it is not surprising that the posterior portion of the left inferior parietal lobule was identified as disconnected from lateral and ventral temporal cortices in association with tool use gesturing performance given its proximity with respect to these white matter tracts.

The strength of our SVR-CLSM approach is that we control for the cumulative amount of node-level disconnection using the results of the SVR-VLSM. Thus, our SVR-CLSM findings are significant over and above cumulative node-level disconnection, total lesion volume, and months post onset and converge with the lesion sites identified in our SVR-VLSM analysis, giving us confidence that we are identifying a robust effect. Moving forward, it will be important to address critiques of multivariate lesion-symptom mapping, including issues regarding statistical control of nonuniform lesion distributions in LCVA, different lesion volume control techniques, and appropriate sample size for multivariate and univariate lesion-symptom mapping analyses (for discussion, see DeMarco and Turkeltaub 2018; Sperber, Wiesen, and Karnath 2019b; Ivanova et al. 2020). In the degree to which convergence across independent analyses can be demonstrated, we can start to develop a common analytic pipeline to control for lesion distribution across datasets. In addition, graph theoretic algorithms like the maximally disconnected subgraph analysis we use offer a data-driven approach to identify nodes that contribute most heavily to cumulative disconnection (see Greene et al. 2019).

Finally, in this study, we focused solely on patterns of disconnection within the left hemisphere. It will be important for future studies to determine the extent to which apraxia severity is associated with interhemispheric disconnection following LCVA. Prior resting-state functional connectivity studies demonstrate that recovery of motor and attentional processes is dependent on the integrity of interhemispheric functional connectivity (He et al. 2007; Carter et al. 2010; Siegel et al. 2016), and recent resting functional connectivity work suggests that interhemispheric disconnection is a significant predictor of apraxia severity (Watson et al. 2019). A combined SVR-CLSM and SVR-VLSM approach provides a rigorous analytic pipeline to further elucidate the degree to which inter- and intra-hemispheric disconnection predicts apraxia severity.

## Conclusion

For decades, the theoretical consensus has been that the left inferior parietal lobule is the locus of manipulation knowledge, as lesions to this site are associated with apraxia. Our results offer a novel interpretation of the role of the left inferior parietal lobule in tool use: Tool manipulation knowledge is not “stored” local to the left inferior parietal lobule; rather, the left inferior parietal lobule is “hub-like” in function, as it aggregates 1) knowledge of action and object representations processed in ventral and lateral posterior temporal cortices with 2) online visuomotor mechanisms supporting the programming of hand and arm actions, and 3) top–down feedback via frontal and motor structures to support the selection and implementation of tool use actions on the basis of task goals, rules, or context. Apraxia can arise from frank parietal damage or due to damage of the white matter interfacing nodes of the Tool Use Network. It will be important for future studies to combine lesion-symptom mapping techniques with structural and functional connectivity measures to test causal hypotheses of the relative contributions to praxis made by distributed action and object representations in the human brain.

## Supplementary Material

GarceaColleagues_ActionDiscon_SOM_Revision_20200710_tgaa035

## Footnotes

1 We regressed variability in months post stroke onset from edge-wise disconnectivity values, given that there was a moderate correlation between total lesion volume and months post onset (*r*(55) = 0.22, *P* = 0.10). Note that months post onset neither correlated with tool use gesturing performance (*r*(55) = 0.09, *P* = 0.52) nor meaningless gesture imitation performance (*r*(55) = −0.07, *P* = 0.60).

## Notes

We thank Cortney Howard, Leyla Tarhan, Louisa Smith, Rachel Metzger, Veronica Kreter, and Harrison Stoll for coding participants’ gestures, and Austin Wild, Olu Faseyitan, and Branch Coslett for help with lesion segmentation. *Conflict of Interest*: None declared.
