# Supplementary material for: Structural Disconnection of the Tool Use Network after Left Hemisphere Stroke Predicts Limb Apraxia Severity
Source: Cereb Cortex Commun. 2020 Jul 28;1(1):tgaa035. doi: 10.1093/texcom/tgaa035 (PMC7573742; doi:10.1093/texcom/tgaa035)
Supplement: GarceaColleagues_ActionDiscon_SOM_Revision_20200710_tgaa035 [file garceacolleagues_actiondiscon_som_revision_20200710_tgaa035.pdf]

Supplemental Materials

Structural Disconnection of the Tool Use Network After Left Hemisphere Stroke Predicts Limb Apraxia Severity

Frank E. Garcea*, Clint Greene, Scott T. Grafton, Laurel J. Buxbaum

*Author to whom correspondence should be addressed.
Email: garceafr@einstein.edu

Supplemental Table 1. Average MNI coordinate of each left hemisphere region in the Lausanne Atlas. Numbers next to each label denote subregion identity.

Region Label	Center of Mass (Mean XYZ)		
Left Lateral Orbitofrontal Cortex 1	-28	23	-18
Left Lateral Orbitofrontal Cortex 2	-19	43	-19
Left Pars Orbitalis	-43	39	-13
Left Frontal Pole	-8	67	-10
Left Medial Orbitofrontal Cortex	-7	39	-18
Left Pars Triangularis	-46	32	1
Left Pars Opercularis	-48	17	14
Left Rostral Middle Frontal Gyrus 1	-36	33	32
Left Rostral Middle Frontal Gyrus 2	-32	49	20
Left Rostral Middle Frontal Gyrus 3	-30	59	-3
Left Superior Frontal Gyrus 1	-9	56	13
Left Superior Frontal Gyrus 2	-8	44	36
Left Superior Frontal Gyrus 3	-17	25	52
Left Superior Frontal Gyrus 4	-10	3	61
Left Caudal Middle Frontal Gyrus	-36	11	47
Left Precentral Gyrus 1	-17	-21	67
Left Precentral Gyrus 2	-34	-16	61
Left Precentral Gyrus 3	-46	-6	43
Left Precentral Gyrus 4	-52	3	19
Left Paracentral Gyrus	-7	-29	59
Left Rostral Anterior Cingulate Cortex	-6	38	1
Left Caudal Anterior Cingulate Cortex	-5	20	27
Left Posterior Cingulate Cortex	-5	-18	38
Left Isthmus Cingulate Cortex	-7	-45	20
Left Postcentral Gyrus 1	-22	-34	70
Left Postcentral Gyrus 2	-44	-27	54
Left Postcentral Gyrus 3	-56	-14	26
Left Supramarginal Gyrus 1	-54	-28	24
Left Supramarginal Gyrus 2	-55	-44	38
Left Superior Parietal Lobule 1	-30	-46	60
Left Superior Parietal Lobule 2	-24	-61	56
Left Superior Parietal Lobule 3	-16	-81	39
Left Inferior Parietal Lobule 1	-42	-77	22
Left Inferior Parietal Lobule 2	-40	-65	40
Left Precuneus 1	-7	-49	53

Left Precuneus 2	-9	-64	30
Left Cuneus	-5	-81	18
Left Pericalcarine Cortex	-11	-82	5
Left Lateral Occipital Cortex 1	-18	-98	6
Left Lateral Occipital Cortex 2	-38	-83	-1
Left Lingual Gyrus 1	-11	-78	-8
Left Lingual Gyrus 2	-17	-57	-2
Left Fusiform Gyrus 1	-37	-65	-14
Left Fusiform Gyrus 2	-34	-27	-28
Left Parahippocampal Gyrus	-25	-31	-19
Left Entorhinal Cortex	-25	-5	-33
Left Temporal Pole	-29	13	-38
Left Inferior Temporal Gyrus 1	-49	-14	-35
Left Inferior Temporal Gyrus 2	-54	-53	-12
Left Middle Temporal Gyrus 1	-61	-47	0
Left Middle Temporal Gyrus 2	-56	-8	-23
Left Bank of the Superior Temporal Sulcus	-53	-46	8
Left Superior Temporal Gyrus 1	-57	-37	14
Left Superior Temporal Gyrus 2	-52	-2	-12
Left Transverse Temporal	-45	-22	9
Left Insula 1	-37	-12	5
Left Insula 2	-35	12	-4
Left Thalamus	-12	-18	6
Left Caudate	-13	8	10
Left Putamen	-25	1	-1
Left Pallidum	-19	-3	-2
Left Accumbens Area	-7	9	-9
Left Hippocampus	-25	-23	-14
Left Amygdala	-24	-3	-21

Supp. Fig. 1. Edge-based Structural Disconnection Overlap among the 57 LCVA Participants.

A. Edge-level Disconnection Overlap.

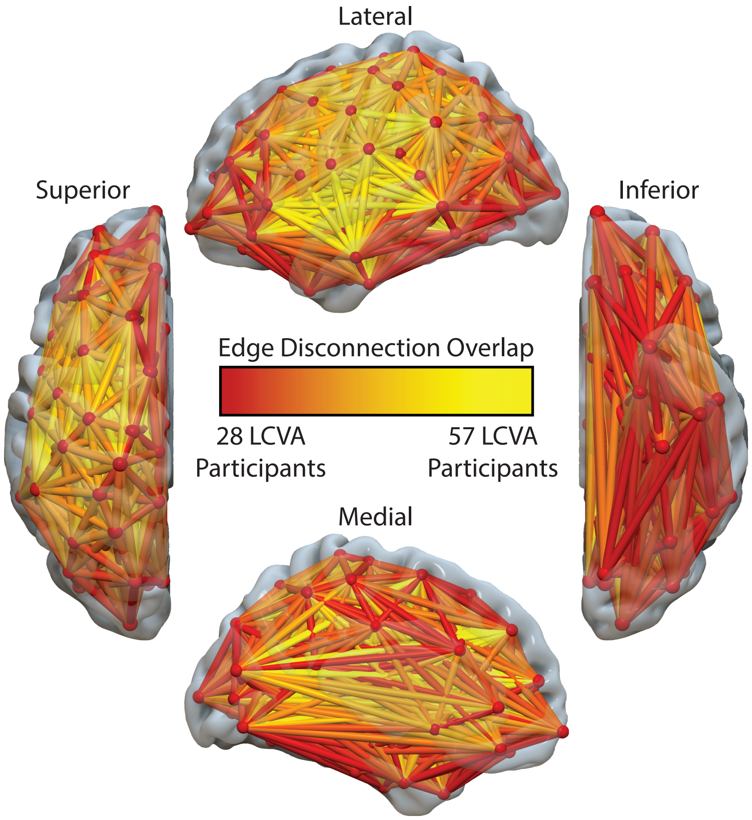

Supplemental Figure 1. Edge-based Structural Disconnection Overlap among the 57 LCVA Participants. A. Edge-based overlap among 57 participants. Only voxels with at least 28 participants are plotted. Note there is maximal disconnection throughout the left hemisphere.

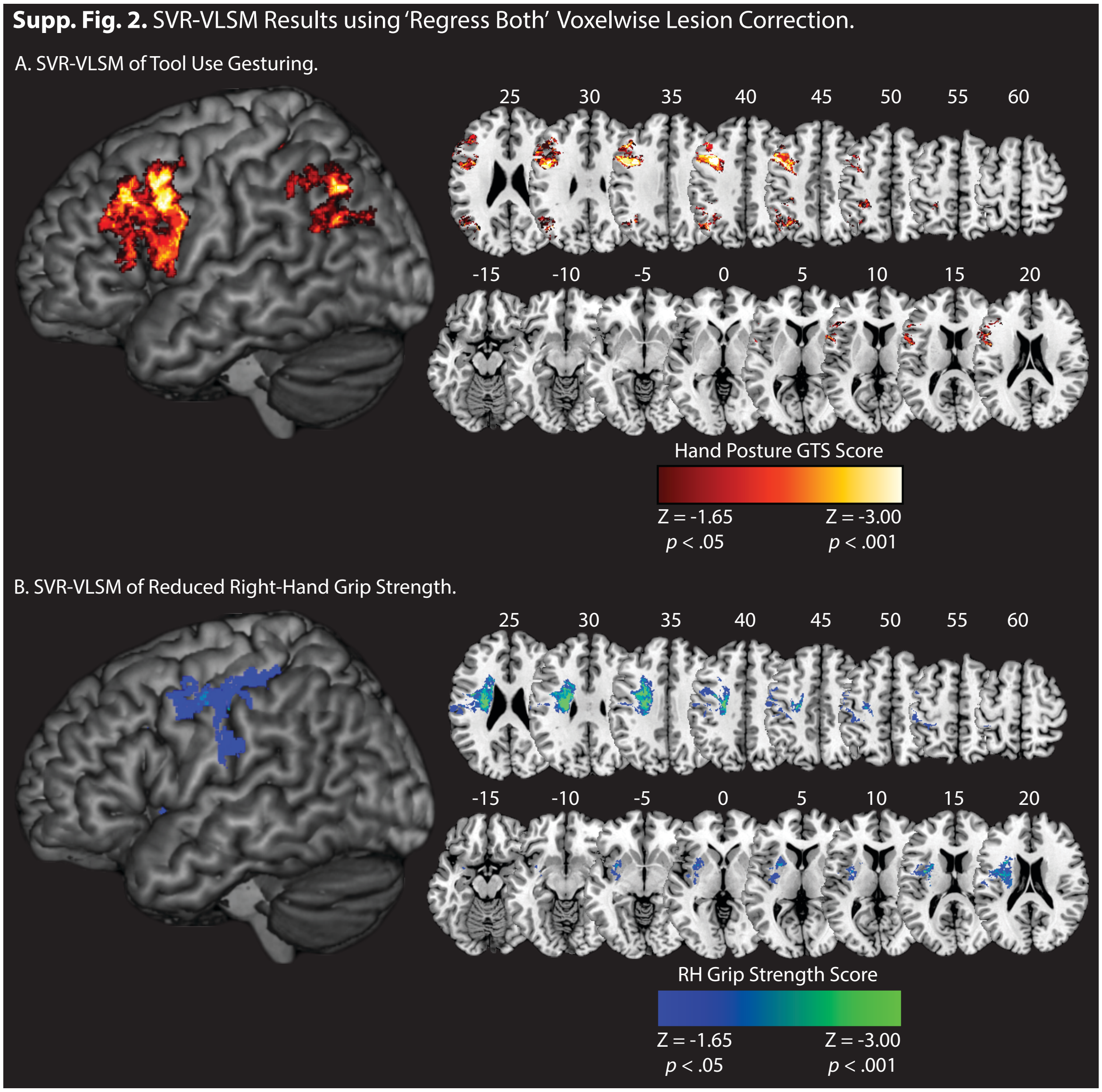

Supplemental Figure 2. SVR-VLSM Results using 'Regress Both' Voxelwise Lesion Correction. A. Voxels associated with hand posture errors in tool use gesturing (greater hand posture errors in tool use gesturing controlling for hand posture errors in meaningless imitation; red-to-white scale) using the 'Regress Both' lesion volume correction. We further removed significant voxels that did not form a cluster of at least 100 contiguous $1mm^3$ voxels. The analysis identifies two clusters. The first is in the left supramarginal gyrus, and includes posterior superior temporal gyrus, the angular gyrus, and post-central gyrus. A second cluster is identified in the left middle frontal gyrus, and overlaps with the left inferior frontal gyrus (pars opercularis), pre-central gyrus, and the posterior portions of pars triangularis. **B.** Voxels associated with reduced right-hand grip strength (weaker grip strength of the right hand controlling for grip strength of the left hand; blue-to-green scale) identify the left pre- and post-central gyri, and subcortical regions including the insula, basal ganglia (caudate, putamen), portions of the frontal operculum, and the superior corona radiata. Whole-brain results are rendered in MNI space in 5-mm increments. SVR-VLSM maps are set to a voxelwise threshold of $p < .05$ with 10,000 iteration Monte Carlo style permutation analysis.

Supp. Fig. 3. SVR-CLSM of Hand Posture Errors in Tool Use Gesturing.

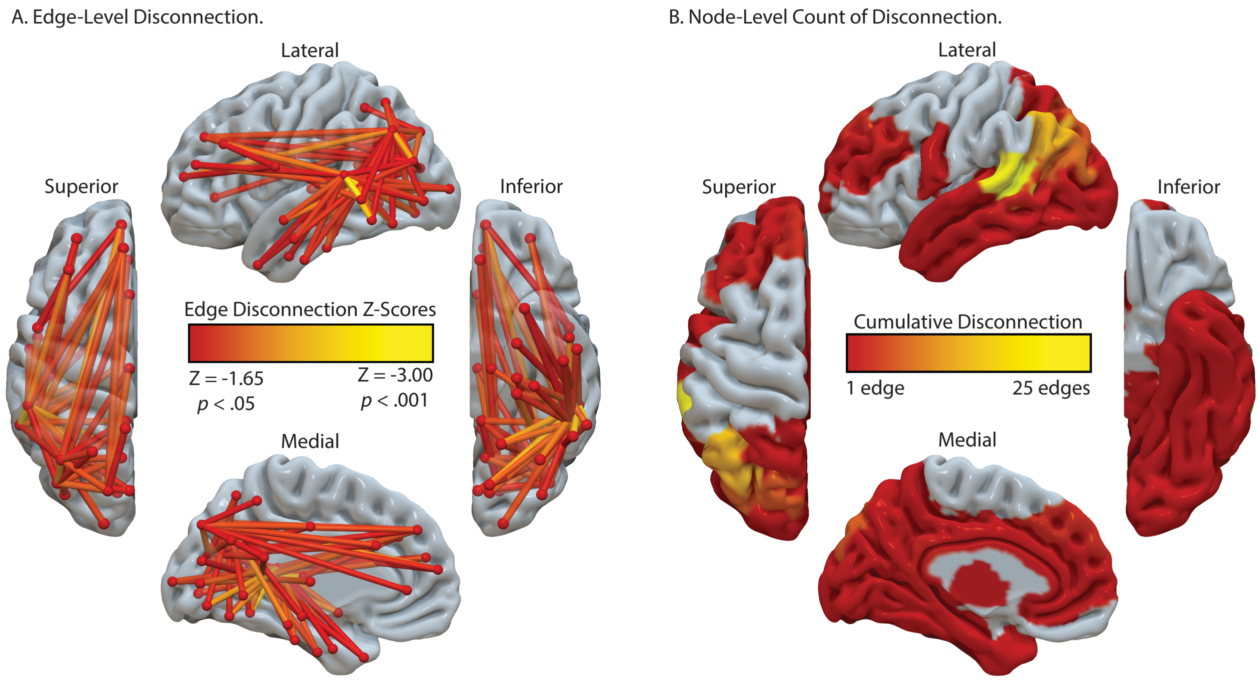

Supplemental Figure 3. SVR-CLSM of Hand Posture Errors in Tool Use Gesturing. A. The result of the SVR-CLSM identifies a disconnected fronto-temporal-parietal network associated with poor tool use gesturing (controlling for meaningless imitation and total lesion volume). Note that these are the significant edges entered into the algorithm that identifies the maximally disconnected subgraph (see Figure 3). **B.** A node-level count of disconnection identifies regions in the left inferior parietal lobule, superior temporal gyrus, middle temporal gyrus, precentral gyrus, and middle and superior frontal gyri as exhibiting increased disconnection in association with hand posture errors in tool use gesturing.

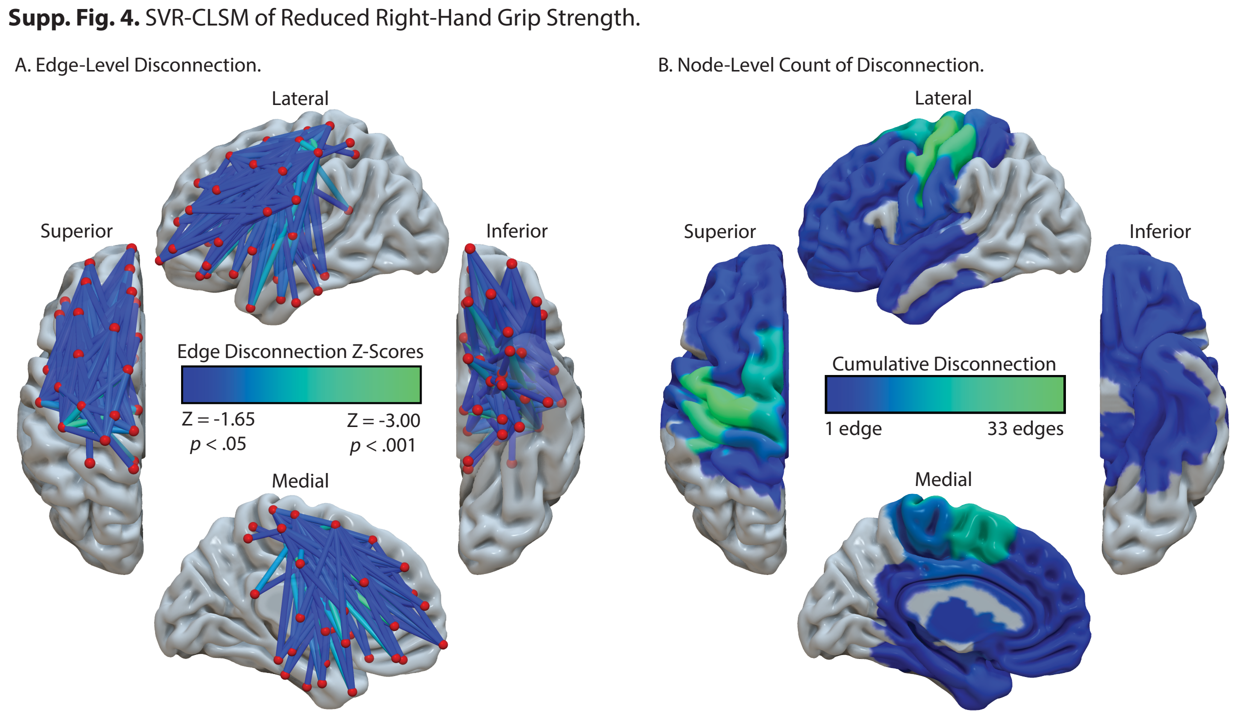

Supplemental Figure 4. SVR-CLSM of Reduced Right-Hand Grip Strength. A. The result of the SVR-CLSM identifies a disconnected fronto-motor network associated with of reduced right-hand grip strength (controlling for variability in left-hand grip strength and total lesion volume). Note that these are the significant edges entered into the algorithm that identifies the maximally disconnected subgraph (see Figure 4). There is increased disconnection between pre- and post-central gyri and subcortical regions, consistent with the results of the SVR-VLSM (Figure 2C), as well as disconnection that includes prefrontal regions. **B.** A node-level count of disconnection identifies regions in the pre- and post-central gyri, and superior frontal gyrus (~ dorsal premotor cortex) as exhibiting increased and widespread disconnection to prefrontal cortex and subcortical motor areas in association with reduced right-hand grip strength.

Supp. Fig. 5. Anatomical Proximity between the left posterior inferior parietal lobule and left SLF.

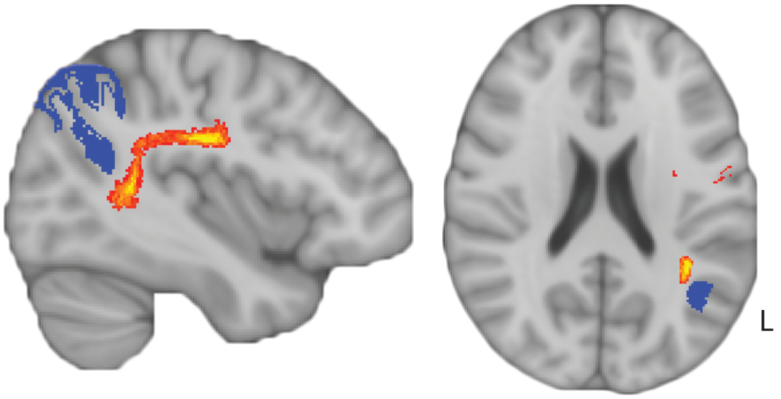

Supplemental Figure 5. Anatomical proximity between the left posterior inferior parietal lobule and the left superior longitudinal fasciculus. Voxels of the left posterior inferior parietal lobule (blue) are identified as structurally disconnected in association with reduced tool use gesturing performance and are situated in close anatomical proximity to the posterior portion of the left superior longitudinal fasciculus (SFL; red-to-yellow). The SLF is defined by the Johns Hopkins White Matter Atlas in FSL, set to a minimum probability value of 20% (red) and a maximum value of 50% (yellow).

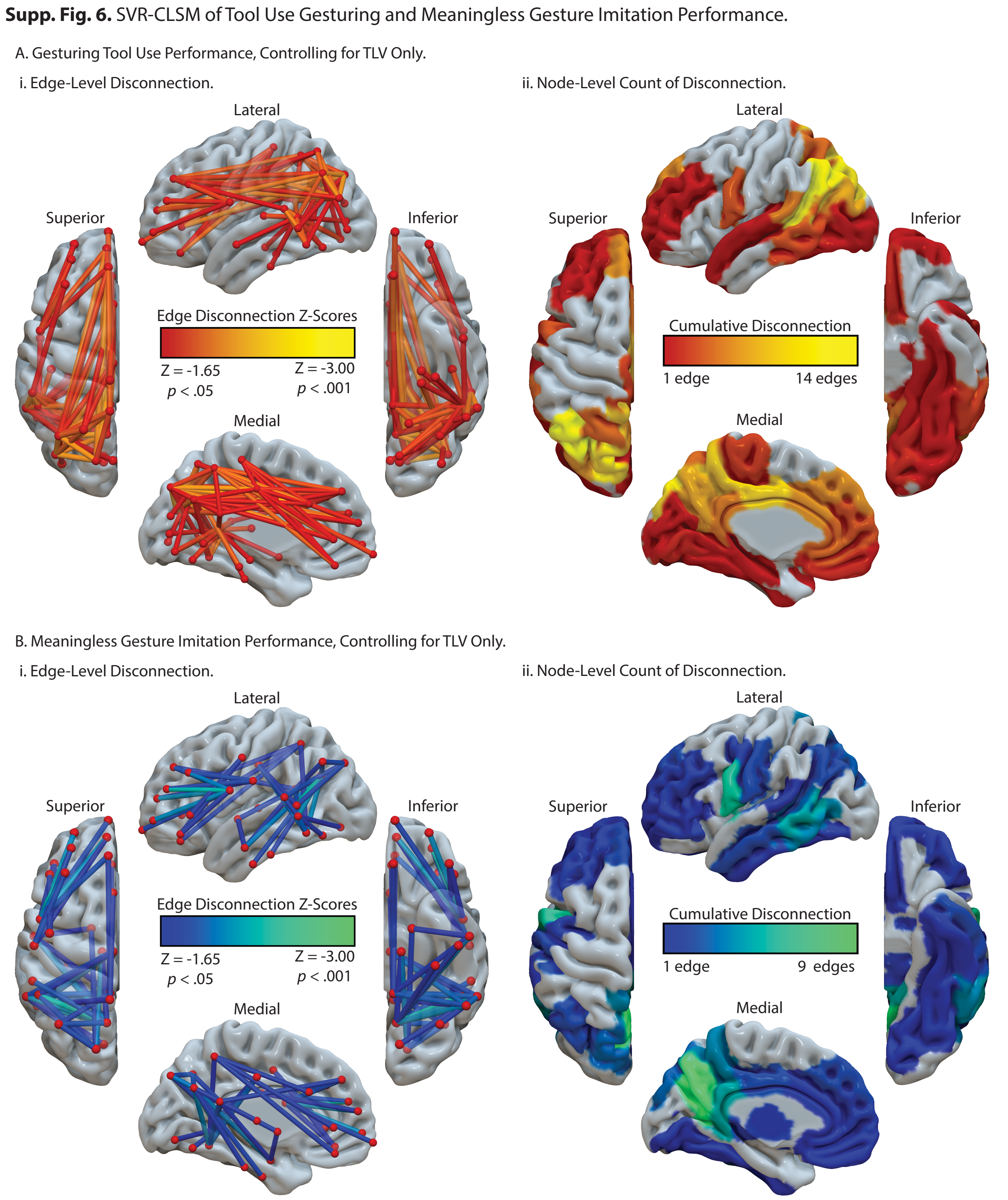

Supplemental Figure 6. SVR-CLSM of Tool Use Gesturing and Meaningless Gesture Imitation Performance. Ai. An SVR-CLSM analysis of tool use gesturing performance, controlling for total lesion volume, identifies a common fronto-temporal-parietal network as was observed when also controlling for performance in meaningless gesture imitation (Supplemental Figure 3). **Aii.** A node-level count of disconnection identifies the left inferior parietal lobule, superior temporal sulcus, and superior and posterior parietal nodes as the regions most

disconnected in association with tool use gesturing performance. **Bi.** An SVR-CLSM analysis of meaningless gesture imitation performance, controlling for total lesion volume, identifies three disconnection clusters: The first is a frontal cluster involving disconnection among inferior, middle, and superior frontal gyri and precentral gyrus; the second is a superior and medial parietal cluster that includes precuneus, the superior and inferior parietal lobule, and superior frontal gyrus; the third is a cluster that includes posterior and superior parietal lobule and ventral and lateral temporal cortex. **Bii.** A node-level count of disconnection identifies the left superior parietal lobule, precuneus, precentral gyrus, and lateral temporal cortex as regions most disconnected in association with meaningless gesture imitation performance.
